# The Roles of Lifetime Enacted Stigma in Tic Symptoms among Young Adults with Tourette Syndrome

**DOI:** 10.1002/mdc3.13900

**Published:** 2023-11-03

**Authors:** Chengshi Shiu, Wei‐Ti Chen, Boram Kim, Emily Ricketts, Jordan T. Stiede, Flint M. Espil, Matthew W. Specht, Douglas W. Woods, John Piacentini

**Affiliations:** ^1^ Department of Social Work and Taiwan Social Resilient Research Center National Taiwan University Taipei Taiwan; ^2^ School of Nursing University of California Los Angeles California USA; ^3^ Department of Psychology Marquette University Milwaukee Wisconsin USA; ^4^ Department of Psychiatry and Behavioral Sciences Baylor College of Medicine Houston Texas USA; ^5^ Department of Psychiatry and Biobehavioral Sciences University of California Los Angeles California USA; ^6^ Department of Psychiatry and Behavioral Sciences Stanford University Stanford California USA; ^7^ Department of Psychiatry Weill Cornell Medicine New York New York USA

**Keywords:** young adults, Tourette syndrome, tic symptoms, enacted stigma, secondary data analysis

## Abstract

**Background:**

Although rarely framed as enacted stigma, adults with Tourette syndrome (ATS) have long suffered from discrimination associated with their tic symptoms. Given the high stress levels of enacted stigma that ATS experience, it is expected that their tic symptoms are profoundly impacted. However, the evidence linking enacted stigma to ATS's tic symptoms remains limited.

**Methods:**

This study used a secondary data‐analysis approach to reanalyze the data from the follow‐up phase of a multi‐centered, randomized controlled trial in which a behavioral intervention was tested for its efficacy in managing tic symptoms. This study first conducted psychometric testing on a list of 16 enacted stigma events across five life stages and identified the underlying factor structure. The Yale Global Tic Severity Scale (YGTSS) was used to assess severity and impairment of current tic symptoms, whereas the Clinical Global Impression of Severity scale (CGI) was used to obtain the gestalt of clinical judgment on tic severity. A series of multivariate linear models were then fitted to test the relationships between different types of lifetime enacted stigma and current tic symptoms.

**Results:**

The analytic sample included 73 young ATS (average age of 23.2 [standard deviation = 2.5] years). The factor analysis identified three types of enacted stigmas: “traumatic events,” “confrontations,” and “subtle mistreatments.” In multivariate models, traumatic events significantly associated with YGTSS‐severity, whereas subtle mistreatments provided additional explanations for CGI.

**Conclusions:**

Enacted stigma may play important roles in shaping ATS's current tics symptom severity and, therefore, should be carefully considered in future intervention development.

## Introduction

Tourette syndrome (TS) impacts 0.01% (0.002%–0.07%)^1^ of the adult population, although the complex developmental course and high heterogeneity in measuring TS symptoms have rendered accurate estimations impossible. Prevalence of TS among school‐age children ranges from 0.32% to 0.85%.^2^ Although many children with TS experience a decrease in frequency and intensity of their TS symptoms over time,^3^ studies show that approximately two thirds experience symptoms that persist into adulthood,^4^ and that 25% of those children have moderate to severe symptoms.^5^

Stigma, as Goffman^6^ conceptualized in his seminal work, refers to an “attribute that is deeply discrediting”, connecting discernible traits with negative social evaluations that lead to reduced “life chances” for the person being stigmatized. The research community has identified several mechanisms through which stigma impacts individuals, including public stigma, enacted stigma, anticipated stigma, and internalized stigma,^7^, ^8^, ^9^ among which, enacted stigma has emerged as one of the most salient dimensions of stigma.^7^, ^10^ Enacted stigma encompasses episodes of discriminatory events based on perceived lower status and group memberships.^11^ Discriminatory acts range from traumatic incidents to subtle mistreatments. Studies show that enacted stigma and discrimination can damage the mental health of the stigmatized.^12^, ^13^, ^14^ Although traumatic incidents may cause post‐traumatic stress disorder in the affected, microaggressions over time can elevate depressive and anxiety symptoms, decreasing mental well‐being.^15^, ^16^ Several theorists identify enacted stigma as one of the key stressors experienced by marginalized individuals,^17^, ^18^, ^19^ not only by altering cognitive‐affective‐behavioral regulation patterns, but also by triggering psycho‐neuro‐immunological sequelae that eventually lead to physical conditions.^19^, ^20^, ^21^

Children with TS have higher risks of becoming a victim of bullying, resulting in diminished self‐esteem, unhealthy self‐concept, and low quality of life.^22^, ^23^, ^24^ Analysis of the United States (US) National Survey of Children's Health data estimated that 56% of youth with TS experienced at least one episode of bullying in the prior year.^25^ Although rarely framed as stigma, adults with Tourette syndrome (ATS) have long suffered from enacted stigma. Available data suggest that ATS constantly experience mistreatment in their daily lives. The Tourette Syndrome Impact Survey (TSIS, 2013) of 672 ATS in the United States found that 68% of respondents suffered discriminatory events, with 30% treated disrespectfully in shops, 17% kicked out of public spaces, 12% treated poorly by their landlords, and 9% lost their jobs.^26^ In the TSIS survey of 2022, 72% of those surveyed reported being discriminated against and 55% believed tics prevented them from reaching their fullest potential.^27^ A recent United Kingdom (UK) survey of 167 ATS^28^ found 54.3% of respondents were “avoided or shunned by people” and more than 50% of respondents experienced enacted stigma in nine of 21 life areas, including “making or keeping friends” (68.3%), “dating or intimate relationships” (57.3%), “family” (57.8%), “neighborhood” (55.3%), “social life” (71.4%), “education” (75.4%), “finding a job” (54.3%), and “public transportation” (60.8%).” In summary, enacted stigma has substantially reduced the “life chances” of ATS.

Involuntary tic symptoms, together with other clinical and social factors, are drivers of enacted stigma experiences. As tic symptoms often violate social norms, they can be perceived unfavorably (eg, as the effects of substance use, psychosis, intellectual disability, aggression) by surrounding people.^29^, ^30^, ^31^ Tic symptoms experienced by ATS are often complex and dynamic, and can be affected by internal and external environments;^32^, ^33^ For example, it has been well documented that stress levels are related to the severity of tic symptoms and even associated with the onset of TS symptoms.^32^, ^34^, ^35^ As such, enacted stigma may contribute to the sustainment or exacerbation of tic symptoms. However, limited studies have empirically tested relationships between different types of enacted stigma and ATS's tic symptoms, hindering our efforts to develop interventions for ATS to better manage their tic symptoms, mental health, and social relationships.

This study investigates the relationships between different types of enacted stigma and tic symptoms in young ATS. We hypothesize that ATS encounter various types of enacted stigma over their lifetime and that enacted stigmas are associated with their tic symptom severity.

## Methods

Secondary data analysis was used to re‐analyze follow‐up data from a multi‐centered, randomized controlled trial (RCT)^36^ that tested the efficacy of comprehensive behavioral intervention for tics (CBIT), a multi‐element treatment package encompassing habit reversal training, function‐based assessment and intervention, relaxation training, and behavioral rewards.^37^ for reducing tic symptoms among children with TS across three urban study sites in the United States. Children, between 9 and 17 years of age, were eligible to participate if they had TS or a chronic tic disorder of moderate or greater severity, an intelligent quotient (IQ) >80, and English fluency. In total, 126 children with TS were enrolled and randomized in a 1:1 ratio. In the control arm, supportive psychotherapy and psychoeducation were offered. Outcomes, captured by the Yale Global Tic Severity Scale (YGTSS) and the Clinical Global Impressions‐Improvement scale, were assessed at baseline, week 5, week 10, month 3, and month 6. The current study dataset was derived from the follow‐up study of the RCT.^38^ A total of 80 participants (63.4%) completed the long‐term assessment administered by trained clinical evaluators through in‐person or videoconference‐based interviews, with an average of 11.2 years between the RCT's conclusion and the follow‐up evaluation. Participants who were lost to follow‐up (23.8%) or declined (12.7%) to participate did not differ significantly from those who participated with regards to their severity and impairment of tic symptoms, mental illness, demographics, treatment assignment, or comorbidity.^38^ The current study selected those who maintained their TS diagnosis (n = 76) and completed the long‐term assessment, resulting in a total of 73 participants in our analytic sample.

### Measurements

#### Enacted Stigma

Enacted stigma was measured by a set of 16 items that were asked repeatedly in reference to five different stages of life. The overall question was, “[w]hen tics were at their worst (during middle school, 9th–10th grade, 11th–12th grade, beginning of college/work, past month), how did other people respond to your tics?” Sixteen different events were listed on the survey for each life stage. Items were developed from the RCT study, covering a wide range of discriminatory events commonly shared by the TS youth and young ATS. Events included “teased”, “told to stop”, and “stared at.” Events that happened during a specific life stage were coded “1” for that item. For each of the 16 items, binary responses across five periods were summed, creating an index that represented lifetime accumulated experiences in relation to current tic symptoms. The theoretical range for each item was between 0 (never happened before) to 5 (happened in all periods). The enacted stigma questions were presented in Appendix S1.

Because the survey included 16 items measuring enacted stigma, exploratory factor analysis was conducted to identify a possible underlying factor structure that would represent all the information with fewer indicators. The reliability of the subscales and the full instrument was calculated using Cronbach's α and McDonald's ω, respectively. The Mokken scale analysis was used to compute the Loevinger's H coefficient to judge the “scalability” of items in each subscale. If the Loevinger's H was >0.5, the scalability of that scale was considered “strong.”^39^, ^40^

Factor analysis yielded a three‐factor structure, as summarized in Table 1. The three factors were “traumatic events” (3 items), “confrontations” (5 items), and “subtle mistreatments” (4 items), that together explained 90% of the variations. Four items, including “asked to leave family activity,” “sent to bed early,” “asked to leave meal early,” and “not included in family activities” were excluded because of low factor loadings and extremely low counts. The Loevinger's Hs of all three subscales were >0.5. Although the Cronbach's α of the “traumatic events” was 0.57, the Cronbach's αs for both “confrontations” and “subtle mistreatments” equaled 0.76. The McDonald's ω for the entire measurement was 0.73, indicating satisfactory quality of the overall enacted stigma measurement. Finally, summary scores were computed by averaging scores across the items within each dimension of enacted stigma. As such, higher scores indicated more types of events experienced by ATS within each dimension of enacted stigma across life stages. Please note, the scores do not assess the frequency of occurrence of enacted stigma events in ATS's daily lives.

**TABLE 1:** Exploratory factor analysis of enacted stigma items and measurement characteristics

	Subscale 1: traumatic events	Subscale 2: confrontations	Subscale 3: subtle mistreatments
Items	Factor loading	Factor loading	Factor loading
Asked to stop a group sport or activity	0.38		
Asked to leave family activities			
Asked to leave class/meeting		0.52	
Asked to leave school/work entirely	0.69		
Asked to leave restaurant		0.73	
Sent to bed early			
Asked to leave meals early			
Not allowed to participate in a sport/activity		0.77	
Not included in family activities			
Not invited to social events			0.59
People tried not to sit near me			0.79
Teased	0.61		
Told to stop		0.40	
Had arguments about tics		0.66	
People asked unwanted questions			0.65
Stared at			0.69
Proportion of variance explained	0.17	0.25	0.48
Loevinger's H coefficient	0.57	0.52	0.68
Cronbach's α	0.57	0.76	0.76
McDonald's ω		0.73	

#### Yale Global Tic Severity Scale

YGTSS is a clinician‐administered assessment tool to evaluate tic symptoms and their impacts over the past week.^41^ YGTSS includes two subscales: symptom severity and tic‐related impairment. The symptom severity subscale rates motor and vocal tic severity separately across five dimensions (number, frequency, intensity, complexity, and interference), and sums the motor and vocal tics severity to create a total tic severity score (range 0–50), with higher scores indicating greater severity. The tic‐related impairment subscale evaluates disability associated with tic symptoms (range 0–50), with higher scores indicating greater disability. The YGTSS has been widely applied and shown good reliability and validity.^42^, ^43^

#### The Clinical Global Impression Scale (CGI)

The Clinical Global Impression Scale is a clinician‐administered measure to assess the overall clinical severity of the tics,^44^ representing the gestalt of clinical judgment in tics severity and takes into account information from standardized assessments and direct observations. At the end of the follow‐up assessment, the evaluator was asked, “Considering your total clinical experience with this particular population, how mentally ill is the subject at this time?” The clinician rates the participants' severity of the tic illness from “no illness” (1) to “severe illness” (7). Although CGI‐S is a single‐item measure, it has been widely adopted across settings, and has shown high degrees of agreement and converging validity with other multi‐itemed tools.^45^, ^46^, ^47^, ^48^

#### Covariates

To adjust the relationships between enacted stigma and the outcomes, covariates were selected as control variables and included demographic attributes, comorbidity, and study design factors. Demographic variables included age (continuous variable), race and ethnicity (non‐Hispanic White, non‐Hispanic all the other races, and Hispanic), sex (female and male), and educational attainment (high school or less, some college, and college or higher). Comorbidity was represented by an index that summed 21 diagnoses, including heart murmur, renopathy, gastrointestinal disease, psychiatric conditions (excluding obsessive‐compulsive disorder), neurological conditions (excluding attention‐deficit/hyperactivity disorder and tics), and hepatic diseases. The study design factors were intervention assignments (intervention and control) and study sites.

### Statistical Analysis

To define the sample, univariate statistics, mean values, standard deviations (SDs), and frequencies were generated. To test the pairwise simple relationships between the selected variables, bivariate analysis was conducted using Pearson correlation coefficient (γ). To better estimate the relationships between enacted stigmas and the outcomes, a multivariate linear model was used where YGTSS‐Total, YGTSS‐Impairment, and CGI were modeled as the outcomes, simultaneously. This approach was used because the outcomes were highly correlated and if modeled separately, the inter‐correlation structure of the three outcomes would be missed, resulting in underestimates of standard errors and smaller confidence intervals.

To evaluate the relationships of enacted stigmas and the outcomes without or without covariates, we built 4 models. Model 1: simple multivariate linear models with traumatic events, confrontations, and subtle mistreatments in separate models without covariates; model 2: multivariate multivariable linear models with traumatic events, confrontations, and subtle mistreatments in separate models with covariates; model 3: multivariate multivariable linear model with traumatic events, confrontations, and subtle mistreatments in the same model without covariates; and model 4: multivariate multivariable linear model with traumatic events, confrontations, and subtle mistreatments in the same model with covariates. The covariates entered in models 2 and 4 included age, race, education, comorbidity, and study design factors. To test whether lifetime enacted stigma provide additional explanations in tic‐related impairments and overall tic severity beyond YGTSS‐Severity, YGTSS‐Severity was added as an independent variable in addition to traumatic events, confrontations, subtle mistreatments and the covariates in model 5. To ensure the quality of the statistical analyses, multivariate normality of the residuals and their homoscedasticity were checked. All analyses were conducted using the Stata 16 statistical package.

## Results

Results of the univariate analysis are summarized in Table 2. The sample was made of 73 young ATS, with a mean age of 23.2 years old (SD = 2.5). More than 80% of the sample was non‐Hispanic White, more than 70% were male, and more than 75% had at least some college. On average, individuals had 2.0 other physical diagnoses (SD = 1.8). Regarding the tic‐related outcomes, on average, participants had an YTGSS‐Total score of 16.5 (SD = 9.7), a YTGSS‐impairment score of 10 (SD = 11.0), and a CGI score of 3.2 (SD = 1.2). This finding suggested that, at the time of the follow‐up assessment, participants experienced mild tic symptoms and mild impairments. Participants also experienced, on average, 0.3 (SD = 0.4) types of traumatic events, 0.3 (SD = 0.4) types of confrontations, and 0.5 (SD = 0.8) types of subtle mistreatments across the five stages of life. As evidenced by the larger SDs of the outcomes and enacted stigmas, a wide variety of tic symptoms, tic‐related impairment, and enacted stigma were experienced among participants.

**TABLE 2:** Sample characteristics and univariate analysis (N = 73)

Variables	Statistics	Theoretical range
Demographic backgrounds		
Age (years), mean (SD)	23.2 (2.5)	19–31
Race, No. (%)		
Non‐Hispanic White	60 (83.3%)	
Hispanic	6 (8.3%)	
Non‐Hispanic all the others	6 (8.3%)	
Sex, No. (%)		
Male	54 (74%)	
Female	19 (26%)	
Education, No. (%)		
HS or less	12 (16.4%)	
Some college	32 (43.8%)	
College or higher	24 (32.9%)	
Missing	5 (6.9%)	
Comorbidity, mean (SD)	2.0 (1.8)	0–21
Outcomes		
YGTSS‐Total Tic Severity, mean (SD)	16.5 (9.7)	0–50
YGTSS‐Impairment, mean (SD)	10 (11.0)	0–50
CGI, mean (SD)	3.2 (1.2)	1–7
Types of enacted stigma		
Traumatic events, mean (SD)	0.3 (0.4)	0–5
Confrontations, mean (SD)	0.3 (0.4)	0–5
Subtle mistreatments, mean (SD)	0.5 (0.8)	0–5

Abbreviations: SD, standard deviation; HS, high school.

Results of the bivariate analysis are presented in Table 3. The three tic‐related outcomes were highly correlated, with Pearson correlation coefficients (γ) all >0.70 (*P* < 0.01). The three types of enacted stigma were moderately correlated, especially so between traumatic events and confrontations (γ=0.40, *P* < 0.01). However, subtle mistreatments had slightly lower correlations with traumatic events (γ=0.35, *P* < 0.01) and with confrontations (γ=0.25, *P* < 0.05). Importantly, all three types of enacted stigma had significant moderate bivariate correlations with all three outcomes, with γ ranging from 0.27 to 0.48. Some selected covariates were significantly correlated with the outcomes and enacted stigma—age and comorbidity correlated positively with all outcomes and traumatic events, suggesting that when the participants were older and had more comorbidities, they also had more severe tic symptoms, greater impairment, and more instances of traumatic events during their lifetime. Being Hispanic was positively associated with more traumatic events, yet, being “non‐Hispanic all the other races” was negatively associated with impairments.

**TABLE 3:** Bivariate analysis among key variables using Pearson correlation

	YGTSS‐ Total Tic Severity	YGTSS‐Impairment	CGI	Traumatic events	Confrontations	Subtle mistreatments	Age	Non‐Hispanic White	Hispanic	Non‐Hispanic all the others	Sex	Education	Comorbidity
YGTSS‐Total Tic Severity	1												
YGTSS‐Impairment	0.73***	1											
CGI	0.87***	0.76***	1										
Traumatic events	0.48***	0.40***	0.45***	1									
Confrontations	0.27**	0.36***	0.29**	0.40***	1								
Subtle mistreatments	0.30**	0.33***	0.41***	0.35***	0.25**	1							
Age, years	0.25**	0.31***	0.21*	0.34***	0.04	0.10	1						
Non‐Hispanic White	0.00	0.07	−0.05	−0.22*	−0.04	0.05	−0.04	1					
Hispanic	0.18	0.14	0.25**	0.28**	0.09	0.03	0.05	‐	1				
Non‐Hispanic all the others	−0.18	−0.23**	−0.18	0.01	−0.04	−0.10	0.00	‐	‐	1			
Sex	0.06	0.19	0.18	0.03	0.05	0.00	0.13	0.17	−0.06	−0.17	1		
Education	0.00	−0.11	0.00	−0.01	−0.12	−0.03	0.28**	−0.03	0.02	0.02	0.09	1	
Comorbidity	0.29*	0.39***	0.30***	0.32***	0.22*	0.22*	0.47***	0.04	0.03	−0.08	0.22†	0.05	1

* *P* < 0.10.

** *P* < 0.05.

*** *P* < 0.01.

Abbreviations: YGTSS, Yale Global Tic Severity Scale; CGI, Clinical Global Impression of Severity scale.

Results of the multivariate analyses are presented in Table 4. Residuals had multivariate normality (Mardia mSkewness = 0.89, Mardia mKurtosis = 14.60, Henze‐Zirkler = 0.93, Doornik‐Hansen = 4.21, all with *P* > 0.10), with no evidence of heteroscedasticity, demonstrating the quality of the multivariate linear models.

**TABLE 4:** Results of multivariate linear models with tic symptoms as the outcomes

	YGTSS‐Total Tic Severity		YGTSS‐Impairment		CGI	
	β coefficients	95% CI	β coefficients	95% CI	β coefficients	95% CI
Model 1						
Traumatic events	12.3***	(6.9–17.7)	11.7***	(5.4–18.0)	1.4***	(0.7–2.1)
	Model *R* ^*2*^ = 0.23		Model *R* ^*2*^ = 0.16		Model *R* ^*2*^ = 0.20	
Confrontations	6.8**	(1.1–12.5)	10.3***	(4.0–16.5)	0.9**	(0.2–1.5)
	Model *R* ^*2*^ = 0.07		Model *R* ^*2*^ = 0.13		Model *R* ^*2*^ = 0.08	
Subtle mistreatments	3.7***	(0.9–6.5)	4.7***	(1.5–7.8)	0.6***	(0.3–0.9)
	Model *R* ^*2*^ = 0.09		Model *R* ^*2*^ = 0.11		Model *R* ^*2*^ = 0.17	
Model 2						
Traumatic events	10.9***	(4.5–17.3)	8.3**	(1.4–15.2)	1.2***	(0.5–2.0)
	Model *R* ^*2*^ = 0.32		Model *R* ^*2*^ = 0.39		Model *R* ^*2*^ = 0.36	
Confrontations	5.7*	(−0.7–12.2)	6.6**	(−0.0–13.1)	0.6	(−0.2–1.3)
	Model *R* ^*2*^ = 0.23		Model *R* ^*2*^ = 0.37		Model *R* ^*2*^ = 0.27	
Subtle mistreatments	2.7*	(−0.5–5.8)	2.9*	(−0.3–6.1)	0.5***	(0.2–0.9)
	Model *R* ^*2*^ = 0.22		Model *R* ^*2*^ = 0.37		Model *R* ^*2*^ = 0.35	
Model 3						
Traumatic events	10.2***	(4.1–16.3)	7.2**	(0.3–14.2)	1.0***	(0.3–1.7)
Confrontations	2.0	(−3.7–7.7)	6.1*	(−0.4–12.6)	0.3	(−0.4–0.9)
Subtle mistreatments	1.7	(−1.0–4.5)	2.7*	(−0.5–5.8)	0.4**	(0.1–0.7)
	Model *R* ^*2*^ = 0.25		Model *R* ^*2*^ = 0.24		Model *R* ^*2*^ = 0.28	
Model 4						
Traumatic events	9.5**	(2.0–17.0)	5.4	(−2.5–13.4)	0.9**	(0.1–1.8)
Confrontations	1.3	(−5.5–8.2)	3.5	(−3.7–10.7)	0.0	(−0.8–0.8)
Subtle mistreatments	1.1	(−2.0–4.2)	1.7	(−1.6–5.1)	0.4**	(0.0–0.8)
	Model *R* ^*2*^ = 0.33		Model *R* ^*2*^ = 0.41		Model *R* ^*2*^ = 0.41	
Model 5						
Traumatic events			−1.0	(−7.5–5.5)	0.0	(−0.5–0.5)
Confrontations			2.6	(−3.0–8.2)	−0.1	(−0.5–3.0)
Subtle mistreatments			1.0	(−1.6–3.6)	0.3***	(0.1–0.5)
YGTSS‐Severity			0.7***	(0.5–0.9)	0.1***	(0.1–0.1)
			Model *R* ^*2*^ = 0.66		Model *R* ^*2*^ = 0.82	

Model 1: simple multivariate linear models with traumatic events, confrontations, and subtle mistreatments in separate models without covariate. Model 2: multivariate multivariable linear models with traumatic events, confrontations, and subtle mistreatments in separate models with covariates. Model 3: multivariate multivariable linear model with traumatic events, confrontations, and subtle mistreatments in the same model without covariate. Model 4: multivariate multivariable linear model with traumatic events, confrontations, and subtle mistreatments in the same model with covariates. Model 5: multivariate multivariable linear model with traumatic events, confrontations, subtle mistreatments and YGTSS‐Severity in the same model with covariates. The covariates entered in the Models 2, 4, and 5 included age, race, education, comorbidity, and study design factors. Note. For clarity, the β coefficients of covariates have been left out.

* *P* < 0.10.

** *P* < 0.05.

*** *P* < 0.01.

Abbreviations: YGTSS, Yale Global Tic Severity Scale; CGI, Clinical Global Impression of Severity scale; CI, confidence interval.

In models 1 and 2, the individual relationships between each enacted stigma with the outcomes were considered. In model 1, where a simple linear model was fitted without any other covariates, all three enacted stigmas significantly associated with the outcomes. However, with only one enacted stigma in each model without any other covariates, enacted stigma can still provide substantial explanation for the outcomes, with the model $R^{2}$ ranging from 0.07 to 0.23. In model 2, where only one enacted stigma was included with the selected covariates, some relationships between enacted stigma and the outcomes were reduced and no longer significant at the 0.05 level. Although traumatic events can still significantly associate with all three outcomes, after controlling for the covariates, confrontations can only significantly associate with YGTSS‐Impairment (adjusted β=6.6, *P* < 0.05), whereas subtle mistreatment can only significantly associate with CGI (adjusted β=0.5, *P* < 0.01).

Models 3 and 4 were considered jointly because the three enacted stigmas had significant moderate correlations and their respective relationships with the outcomes were tested in the presence of other enacted stigmas. In model 3, where only the enacted stigmas were entered into the model without any other covariates, traumatic events emerged as the most powerful predictor for all three outcomes. Whereas, when compared with model 1, the relationships between confrontations and subtle mistreatments and the outcomes were largely reduced and no longer significant at the 0.05 level. An exception, was the relationship between subtle mistreatments and CGI, where subtle mistreatments still provided significant explanation (adjusted β=0.4, *P* < 0.05) for clinician's judgment of tic severity, in addition to traumatic events. In model 3, all the model $R^{2}$ were close to or >0.25, indicating that the enacted stigmas, when considered together, could explain nearly 25% of the variations in outcomes.

In model 4, where the three enacted stigmas were considered together with all the covariates, the traumatic events remained a significant predictor for YGTSS‐Total Tic Severity (adjusted β=9.5, *P* < 0.05) and CGI (adjusted β=0.9, *P* < 0.05); confrontations did not significantly associate with any of the outcomes and subtle mistreatments significantly associated with CGI (adjusted β=0.4, *P* < 0.05). Although none of the enacted stigmas could significantly associate with YGTSS‐Impairment in model 4, given the model $R^{2}$ and the point estimations of the β coefficients, this lack of significant estimations could be largely attributed to the smaller sample size and insufficient power. Additionally, as the resulting variance inflation factor of the model ranged from 1.34 to 1.77, this lack of significant estimations was unlikely to be because of multicollinearity among the selected variables.

In model 5, where YGTSS‐Total Tic Severity was included as an additional predictor, the YGTSS‐Total Tic Severity was significantly associated with YGTSS‐Total Impairment (adjusted β=0.7, *P* < 0.01) and CGI (adjusted β=0.1, *P* < 0.01). However, subtle mistreatments remained significantly associated with CGI (adjusted β=0.3, *P* < 0.01).

## Discussion

This study aimed to formally test the associations between lifetime enacted stigma and current tic symptoms among young ATS. Using statistical analyses on follow‐up data from a multi‐centered RCT, it was demonstrated that among the 16 listed enacted stigma events summed across five life stages, three factors could explain 90% of the total variations: “traumatic events”, “confrontations”, and “subtle mistreatments.” The multivariate analyses also showed that, although independently the events had different relationships with the outcomes, lifetime traumatic events could associate with current severity of tic symptoms, and lifetime subtle mistreatments could associate with the gestalt of clinical judgment on tic severity, even if all factors are considered.

Evidence to support our first hypothesis, young ATS have experienced various types of enacted stigma over their lifetime, was found. Three broad categories of enacted stigma were identified. Three items formed the variable “traumatic events”: “asked to stop a group sport or activity”, “asked to leave school/work entirely”, and “(being) teased.” It is likely that young ATS were removed abruptly from the activities they are actively participating in, were removed completely, or were the targets of verbal attacks—causing very high degrees of distress and setting these stigma events apart from others. In contrast, events that formed “confrontations” for ATS included: removing them from activities they passively participate in (meetings, classes, or restaurants), preventing them from participating in group sports or activities, and having uncomfortable verbal exchanges with others. These events, although still distressing, may not provoke the same levels of distress as traumatic events. However, because these events all involve direct confrontations and marked exclusions, these two types of enacted stigmas do moderately correlate with each other. Four items that formed “subtle mistreatments” included: “not invited to social events”, “people tried not to sit near me”, “people asked unwanted questions”, and “stared at”; events often subtle and conceptually similar to microaggressions.^15^

Although few prior studies collected similar data on types of discriminatory events, all treated those events as mutually independent and somewhat exchangeable, ignoring inter‐correlations among the events, as well as possible underlying structures.^26^, ^27^, ^28^ The three types of enacted stigma identified in this study, all interpersonal in nature, may further develop Malli and Forrester‐Jone's^28^ concept of “stigma in interpersonal relationships” among ATS by distinguishing three different, yet correlated, dimensions of interpersonal stigma.

Some evidence supported our hypothesis that different types of enacted stigma are associated with ATS's tic symptoms differently. In this study, we observed that lifetime traumatic events were independently linked to current tic severity, tic‐related impairments, and clinical assessments of tic severity. On the other hand, lifetime confrontations were associated with current tic‐related impairments, and lifetime subtle mistreatments were linked to clinical evaluations of tic severity. However, when considered together, we found that only lifetime traumatic events were associated with tic severity and clinical assessments of tic severity, while lifetime subtle mistreatments were associated with clinical assessments of tic severity. Given traumatic events potency in generating high degrees of distress, it is expected that young ATS who encounter more lifetime traumatic events may also experience sustained or even worsening tic symptoms. Lifetime effects of traumatic events are quite pronounced, even if they do not occur often. Indeed, traumatic events contributed <20% of the total enacted stigma experiences among the young ATS. Lifetime confrontations, although more frequent, are not as powerful as traumatic events in associating with current tic symptoms. Because confrontations share similar stress‐inducing effects as traumatic events, once traumatic events are considered, confrontations do not provide additional explanations for tic symptom experiences.

Lifetime subtle mistreatments emerged as a robust contributor to the gestalt of clinical judgment on tic severity, although subtle mistreatments did not associate with current tic symptoms, as measured by the YTGSS. Despite the finding that subtle mistreatments typically do not generate as high a degree of stress when compared to traumatic events, because they happen frequently and contribute to nearly 50% of the total enacted stigma experiences, the prolonged, milder stress of subtle mistreatments can take their toll even after their current tic severity is controlled for. This is consistent with the literature on microaggressions. Microaggressions occur almost on a daily basis and serve as a constant reminder of the stigmatization and hostility a person faces in their living environment.^49^ Overtime, the accumulated mild stress may exhaust psychological resources, leading to deteriorated capacity in regulating tic symptoms.

However, the study findings need to be contextualized by limitations. First, our smaller sample size did not provide sufficient power to detect possible relationships, increasing our risk of Type II error. Second, discriminatory events across multiple life stages relied on participant recall, hence rendering the measurement prone to recall biases. Third, the list of enacted stigmas used was by no means comprehensive, only describing interpersonal stigma and not experiences related to perceived lost opportunities such as employment. Fourth, although young ATS interpreted this list of adverse events as enacted stigma in response to their tics, these negative responses may have also been partially triggered by ATS's other conditions, particularly attention‐deficit/hyperactivity disorder or obsessive‐compulsive disorder. Finally, because the data were cross‐sectional in nature, we were unable to ascertain the directions of causality. It is likely that young ATS who have suffered from persistent severe tic symptoms are easy targets for discriminatory events, and hence, reported more types of enacted stigma as a result. Notwithstanding these limitations, this study provides some of the first evidence of its kind to empirically link enacted stigma experienced by young ATS to their current tic symptoms. Additionally, because the data arose from an RCT, the key variables used in this study were systematically assessed by trained clinical evaluators, resulting in high quality of data with lower risk of errors. Future studies may consider adopting prospective study designs with a larger sample and a well‐developed tool to measure TS‐specific stigma.

Individuals with TS have long suffered from enacted stigmas and their consequences. However, a limited number of available interventions carefully consider stigma as a formal component to assist individuals with TS in better managing discriminatory events and symptom ramifications.^50^, ^51^ In light of the results of this study, future interventions may more effectively meet ATS's needs by incorporating principles of trauma‐informed care at both clinical and organization levels and thereby provide ATS a safe environment to reflect on past experiences of enacted stigma and the roles these adverse experiences had in shaping their tic symptoms.

## Author Roles

(1) Research project: A. Conception, B. Organization, C. Execution; (2) Statistical Analysis: A. Design, B. Execution, C. Review and Critique; (3) Manuscript Preparation: A. Writing of the First Draft, B. Review and Critique;

C.S.: 2A, 2B, 2C, 3A, 3B.

W.T.C.: 2A, 2B, 2C, 3A, 3B.

B.K.: 2C, 3B.

E.R.: 2C, 3B.

J.T.S.: 1C, 3B.

F.M.E.: 1A, 1B, 1C, 3B.

M.W.S.: 1A, 1B, 1C, 3B.

D.W.W.: 1A, 1B, 1C, 3B.

J.P.: 1A, 1B, 1C, 2C, 3B.

## Disclosures

**Ethical Compliance Statement**: The authors confirm that it was not required for this secondary data analysis work. However, in the original study, the informed consent was obtained from all the study participants. Furthermore, we confirm that we have read the Journal's position on issues involved in ethical publication and affirm that this work is consistent with those guidelines.

**Funding Sources and Conflicts of Interest**: This research was conducted with support from Tourette Association of America funding to Drs. Woods, Specht, and Piacentini, and National Institute of Mental Health (NIMH) R01MH070802 funding to Dr. Piacentini, and prepared with support from the 2022‐2023 UCLA Faculty Research Grant (PI: Chen, Wei‐Ti), and partial support from the NIH/NIMH P30MH058107 (PI: Steven Shoptaw), and by the Ministry of Education in Taiwan #112L900305. Also, no specific funding was received for this work. The authors declare that there are no conflicts of interest relevant to this work.

**Financial Disclosures for the Previous 12 Months**: The authors declare that there are no additional disclosures to report.

## Supporting information


**Appendix SI.** Enacted stigma was measured by a set of 16‐item questions. The theoretical range for each item was between 0 (*never happened before*) to 5 (*happened in all periods*).
