# Supplementary material for: The Roles of Lifetime Enacted Stigma in Tic Symptoms among Young Adults with Tourette Syndrome
Source: Mov Disord Clin Pract. 2023 Nov 3;10(12):1759–68. doi: 10.1002/mdc3.13900 (PMC10715356; doi:10.1002/mdc3.13900)
Supplement: Supplementary file 1 — Appendix SI. Enacted stigma was measured by a set of 16‐item questions. The theoretical range for each item was between 0 (never happened before) to 5 (happened in all periods). [file MDC3-10-1759-s001.docx]

Supplemental Appendix 1:

Enacted stigma questions

**The manuscript authors retain the copyright.**

**When tics were at their worst during [middle school/junior high; high school 9^th^-10^th^ grade; high school 11^th^-12^th^ grade; beginning of college/work; past month], how did other people respond to your tics? (Check all that apply).**

I Was removed from doing things I wanted to do.

○ Asked to stop a group sport or activity

○ Asked to leave family activities

○ Asked to leave class

○ Asked to leave school entirely

○ Asked to leave restaurant

○ Sent to bed early

○ Asked to leave meals early

○ Other

I Was left out.

○ Not allowed to participate in a sport/activity

○ Not included in family activities

○ Not invited to social events (i.e. birthday parties, social gatherings)

○ People tried not to sit near me

○ Other

I Was given attention.

○ Teased

○ Told to stop

○ Had arguments about tics

○ People asked unwanted questions

○ Stared at

○ Other
